# Controlling coherent perfect absorption via long-range connectivity of defects in three-dimensional zero-index media

**DOI:** 10.1515/nanoph-2023-0485

**Published:** 2023-10-25

**Authors:** Dongyang Yan, Ran Mei, Mingyan Li, Zhikai Ma, Zhi Hong Hang, Jie Luo

**Affiliations:** Institute of Theoretical and Applied Physics, School of Physical Science and Technology, Soochow University, Suzhou 215006, China; State Key Laboratory for Mesoscopic Physics and Frontiers Science Center for Nano-optoelectronics, School of Physics, Peking University, Beijing 100871, China; School of Physical Science and Technology and Collaborative Innovation Center of Suzhou Nano Science and Technology, Soochow University, Suzhou 215006, China; Institute for Advanced Study, Soochow University, Suzhou 215006, China; Provincial Key Lab of Thin Films, Soochow University, Suzhou 215006, China

**Keywords:** coherent perfect absorption, zero-index media, long-range connectivity, absorption control, geometry-invariance

## Abstract

Coherent perfect absorption (CPA), as time-reversed lasing, arises from appropriate wave interference within absorbers, offering flexible control over wave absorption. Typically, this control involves tuning the phase difference between two counter-propagating incident beams. Here, we elucidate the critical role of defect connectivity within three-dimensional zero-index media for realizing and controlling CPA. Specifically, the realization of CPA critically depends on the establishment of long-range connectivity of defects in a specific direction. Once the long-range connectivity is established, the CPA exhibits remarkable resilience against defects’ deformation, changes in size and shape of the zero-index media, as well as variations in number and orientation of incident channels. Notably, a minor disruption to this connectivity will result in a complete reduction of absorption to zero, highlighting an ultra-sensitive absorption property in response to connectivity perturbations. Our findings not only unveil a physical mechanism for realizing CPA but also open up promising avenues for advanced CPA control with versatile functionalities.

## 1 Introduction

Perfect absorption of electromagnetic waves is of great interest and importance across diverse applications including photovoltaics and stealth technologies [1–3]. In the past decade, a unique way to achieve perfect absorption with more than one incident beam, i.e., coherent perfect absorption (CPA) [4–7], has witnessed rapid advancements. Conceptually resembling time-reversed lasers, CPA arises from appropriate wave interference within absorbers. Notably, the CPA scheme surpasses traditional absorption configurations by providing flexible control over wave absorption, allowing continuous adjustment from 100 % to zero absorption through phase difference tuning among incident beams, without modulating intrinsic nonlinearity and absorption coefficient [4–7]. This makes CPA particularly attractive to applications of modulators, switches, and transducers. Up to now, diverse types of CPA implementations have emerged, including the CPA-lasing [8–14], broadband CPA [15–21], and geometry-invariant CPA [22, 23], etc. The geometry-invariant CPA based on zero-index media (ZIM) [24–32] is of special interest. Unlike standard CPA scenarios, where perfect absorption occurs under the illumination of two counter-propagating beams, geometry-invariant CPA allows for more than two incident beams, thereby offering additional degrees of freedom to manipulate wave absorption. However, the previously proposed geometry-invariant CPA schemes primarily rely on two-dimensional (2D) photonic-doped ZIM, which may constrain their practical applications in three-dimensional (3D) space. In addition, the additional dimension-introduced new physics to realize the CPA and extra degrees of freedoms to tune the absorption in 3D models are worth exploring.

Nevertheless, the principle of the geometry-invariant CPA in 2D ZIM is inapplicable to 3D systems due to fundamental differences in electromagnetic wave behaviors. In 2D ZIM, electromagnetic waves follow scalar wave equations, while in 3D ZIM, they obey vector wave equations. Consequently, numerous electromagnetic properties of 2D and 3D ZIM exhibit fundamental distinctions [13, 33], [34], [35], [36]. For instance, the photonic doping effect [37–42], which serves as the basis for geometry-invariant CPA in 2D ZIM, is absent in 3D ZIM. Instead, unusual phenomena like electromagnetic wave percolation [33] and photonic antidoping effect [13, 34] are observed in 3D ZIM when embedded with defects. However, these phenomena cannot be exploited to achieve CPA, as the embedded defects do not affect the absorption at all. Therefore, novel physical mechanisms must be explored to realize the geometry-invariant CPA based on 3D ZIM.

**Figure 1:**
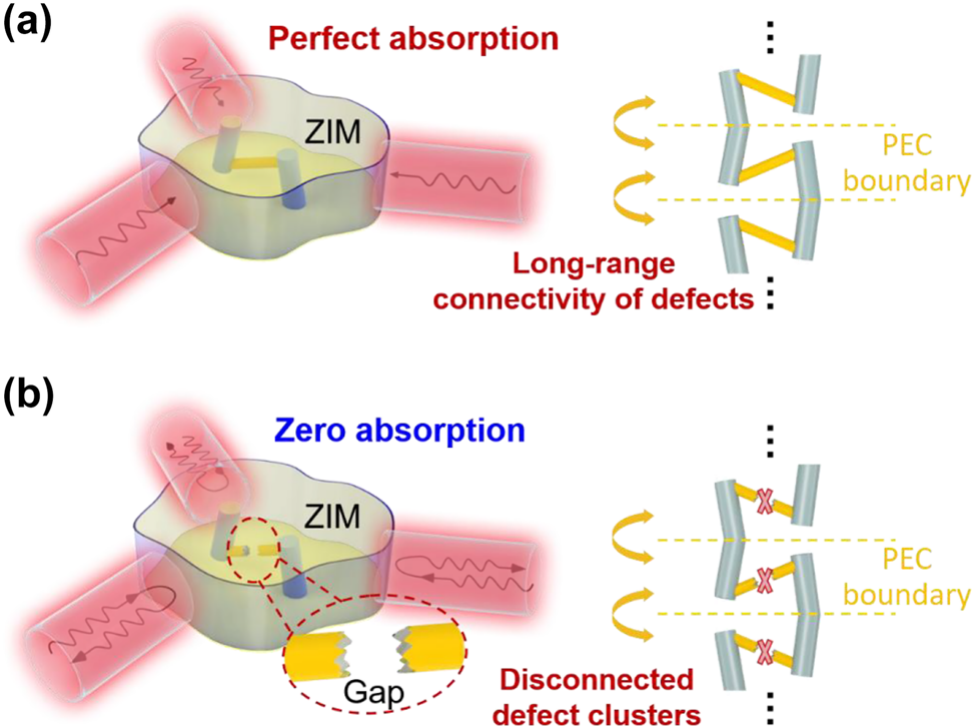
Schematic graphs of geometry-invariant three-channel CPA based on a 3D ZIM embedded with absorptive defects. Such CPA is critically sensitive to the establishment of long-range connectivity of defects in the vertical direction. (a) The defect cluster forms a continuous connection between the upper and lower PEC plates. Since PEC boundaries can be viewed as mirror symmetry planes, the long-range connectivity of defects in the vertical direction is established, in which case CPA is obtained. (b) A minor disruption, such as a small gap, in the defect connectivity will break the long-range connection, and mirrored defects will form separated clusters that are disconnected with each other in the vertical direction. In this case, there is no absorption.

In this work, we elucidate the critical role of defect connectivity within 3D ZIM for achieving and controlling the CPA. We demonstrate that the successful realization of CPA crucially relies on the establishment of long-range connectivity of defects in a specific direction. Once this long-range connectivity is established, geometry-invariant multichannel CPA can be obtained, which exhibits remarkable resilience against defects’ deformation, changes in size and shape of the ZIM, as well as variations in number and orientation of incident channels (Figure 1(a)). Interestingly, even a minor disruption, such as a small gap, will break the long-range connectivity and result in a complete reduction of absorption to zero (Figure 1(b)), highlighting an ultra-sensitive absorption property in response to connectivity perturbations. To practically demonstrate these exceptional properties, a practical design using photonic crystals (PhCs) embedded with conductive films is demonstrated. Our work not only unveils a physical mechanism underlying the realization and control of CPA in 3D ZIM but also opens up possibilities for developing ultra-sensitive sensors.

## 2 Results

### 2.1 Connectivity-controlled CPA and the underlying physics

**Figure 2:**
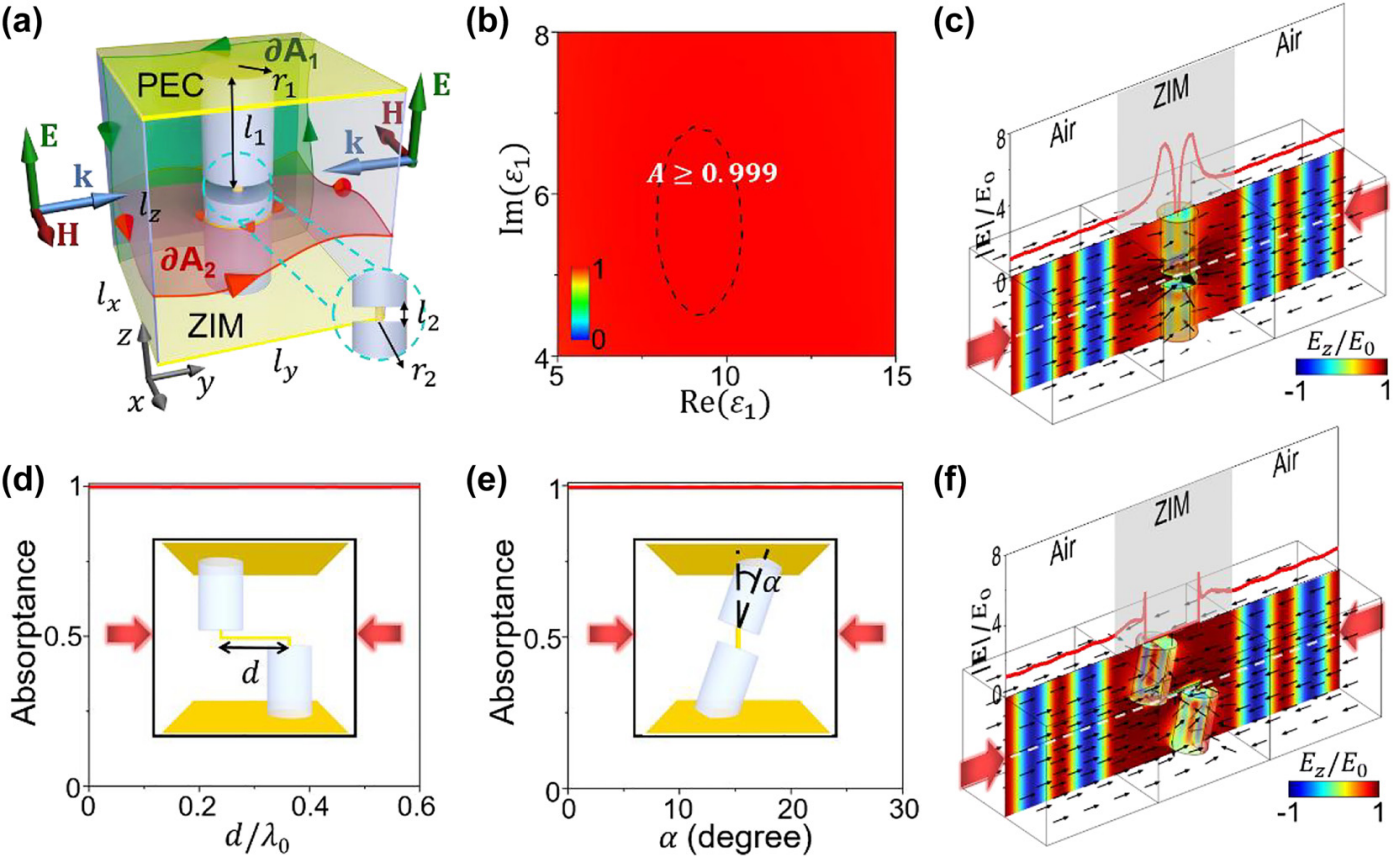
Realization of robust CPA in the presence of long-range connectivity of defects. (a) Schematic graph of a two-channel CPA model consisting of a 3D ZIM slab inside a PEC-PMC waveguide. Inside the ZIM slab, two thick cylindrical defects connecting the upper and lower PEC plates are interconnected by a thin PEC cylindrical defect. Curves ∂*A*_1_ (green) and ∂*A*_2_ (red) represent the boundaries of vertical and horizontal integral surfaces, respectively. Two counter-propagating planer waves with electric fields polarized along the *z* direction are normally incident onto the ZIM slab from free space. (b) Absorptance with respect to Re(*ɛ*_1_) and Im(*ɛ*_1_) of the thick cylinders. (c) Simulated *E*_*z*_/*E*_0_ (color), time-averaged energy flux (arrows), and |**E**|/*E*_0_ (red line) along the white dashed line for the model in (a) with *ɛ*_1_ = 9 + 5.5*i*. (d) Absorptance variation when there is a lateral shift of *d* along the *y* direction between the two thick cylinders. (e) Absorptance variation when the cylinder cluster is rotated by an angle of *α* measured from the *z* axis. In (d) and (e), the upper and lower thick cylinders are always interconnected by the thin PEC cylinder, as illustrated by the insets. (f) Simulated *E*_*z*_/*E*_0_ (color), time-averaged energy flux (arrows), and |**E**|/*E*_0_ (red line) along the white dashed line when changing both the position and orientation of the cylinder cluster.

To explore the physical mechanisms underlying the realization and control of CPA based on 3D ZIM, we adopt a two-channel model for simplicity, as schematically shown in Figure 2(a). The model comprises a 3D ZIM slab (relative permittivity *ɛ*_ZIM_ ∼ 0, relative permeability *μ*_ZIM_ ∼ 0, *l*_*x*_ × *l*_*y*_ × *l*_*z*_ in dimension) embedded with two thick dielectric cylindrical defects (radius *r*_1_, length *l*_1_, relative permittivity *ɛ*_1_), interconnected by a thin perfect electric conductor (PEC) cylindrical defect (radius *r*_2_, length *l*_2_). This ZIM slab is placed inside a PEC – perfect magnetic conductor (PMC) waveguide (cross section *l*_*x*_ × *l*_*z*_ in dimension), which supports transverse electromagnetic waves without cutoff frequencies [43].

We consider two counter-propagating planer waves with electric fields polarized along the *z* direction, incident normally onto the ZIM slab from free space (Figure 2(a)). The two incident waves are in phase with identical amplitude. Their electric and magnetic fields for left (or right) incidence can be expressed as follows: $E_{\text{in}}^{\text{L}}=\hat{z}E_{0}{\text{e}}^{-i\omega t+ik_{0}y}$ and $H_{\text{in}}^{\text{L}}=\hat{x}E_{0}/Z_{0}{\text{e}}^{-i\omega t+ik_{0}y}$ (or $E_{\text{in}}^{\text{R}}=\hat{z}E_{0}{\text{e}}^{-i\omega t-ik_{0}\left(y-l_{y}\right)}$ and $H_{\text{in}}^{\text{R}}=-\hat{x}E_{0}/Z_{0}{\text{e}}^{-i\omega t-ik_{0}\left(y-l_{y}\right)}$ ), where *E*_0_, *ω*, *k*_0_, and *Z*_0_ are the electric field amplitude, angular frequency, wave number in free space, and characteristic impedance of free space, respectively. Due to the infinitely long wavelength within the ZIM arising from *ɛ*_ZIM_ ∼ 0 and *μ*_ZIM_ ∼ 0, only transverse electromagnetic waves are supported in the ZIM region. Consequently, despite the presence of cylindrical defects, the electric and magnetic fields on the left and right *xz* surfaces of the ZIM slab remain nearly uniform. Under this circumstance, we can express the electric and magnetic fields of the backward propagating waves in the left region as follows: $E_{\text{back}}^{\text{L}}=\hat{z}R_{L}E_{0}{\text{e}}^{-i\omega t-ik_{0}y}$ and $H_{\text{back}}^{\text{L}}=-\hat{x}R_{L}E_{0}/Z_{0}{\text{e}}^{-i\omega t-ik_{0}y}$ . These fields are the superposition of the reflected waves from the left incidence and the transmitted waves from the right incidence. Similarly, the electric and magnetic fields of the forward propagating waves in the right region are given by $E_{\text{back}}^{\text{R}}=\hat{z}R_{R}E_{0}{\text{e}}^{-i\omega t+ik_{0}\left(y-l_{y}\right)}$ and $H_{\text{back}}^{\text{R}}=\hat{x}R_{R}E_{0}/Z_{0}{\text{e}}^{-i\omega t+ik_{0}\left(y-l_{y}\right)}$ , respectively. Here, *R*_*L*_ and *R*_*R*_ are two unknown coefficients to be determined.

Next, we apply the Maxwell’s equations, i.e., $\underset{\partial A_{1}}{\oint }E\cdot \text{d}l=i\omega \mu_{\text{ZIM}}\underset{A_{1}}{\iint }H\cdot \text{d}S$ and $\underset{\partial A_{2}}{\oint }H\cdot \text{d}l=-i\omega \varepsilon_{\text{ZIM}}\underset{A_{2}}{\iint }E\cdot \text{d}S$ to the ZIM. Here, ∂*A*_1_ and ∂*A*_2_ represent the boundaries of the vertical integral surface *A*_1_ and horizontal integral surface *A*_2_, as indicated by green and red lines in Figure 2(a), respectively. Due to the properties of *ɛ*_ZIM_ ∼ 0 and *μ*_ZIM_ ∼ 0, the two equations simplify to:

(1a) $$\underset{\partial A_{1}}{\oint }E\cdot \text{d}l=0,$$

and

(1b) $$\underset{\partial A_{2}}{\oint }H\cdot \text{d}l=0.$$

The integral $\oint_{\partial A_{1}}E\cdot \text{d}l$ (or $\oint_{\partial A_{2}}H\cdot \text{d}l$ ) generally comprises two components: an integral around the ZIM denoted as $\oint_{\partial A_{1},Z}E\cdot \text{d}l$ (or $\oint_{\partial A_{2},Z}H\cdot \text{d}l$ ) and an integral around the embedded defects represented as $\oint_{\partial A_{1},D}E\cdot \text{d}l$ (or $\oint_{\partial A_{2},D}H\cdot \text{d}l$ ). Considering the fact that the tangential electric (or magnetic) fields on the PEC (or PMC) boundaries are zero, we obtain $\oint_{\partial A_{1},Z}E\cdot \text{d}l=\left(R_{R}-R_{L}\right)E_{0}l_{z}{\text{e}}^{-i\omega t}$ and $\oint_{\partial A_{2},Z}H\cdot \text{d}l=\left(2-R_{R}-R_{L}\right)E_{0}l_{x}/Z_{0}{\text{e}}^{-i\omega t}$ . Consequently, Eq. (1) can be reformulated as follows:

(2a) $$\underset{\partial A_{1},D}{\oint }E\cdot \text{d}l=\left(R_{L}-R_{R}\right)E_{0}l_{z}{\text{e}}^{-i\omega t},$$

and

(2b) $$\underset{\partial A_{2},D}{\oint }H\cdot \text{d}l=\left(R_{R}+R_{L}-2\right)E_{0}l_{x}/Z_{0}{\text{e}}^{-i\omega t}.$$

The CPA requires that *R*_*R*_ = *R*_*L*_ = 0. In this case, Eq. (2) is simplified to,

(3a) $$\underset{\partial A_{1},D}{\oint }E\cdot \text{d}l=0,$$

and

(3b) $$\underset{\partial A_{2},D}{\oint }H\cdot \text{d}l=-2E_{0}l_{x}/Z_{0}{\text{e}}^{-i\omega t}.$$

Equation (3) presents the essential condition for CPA, which holds for any kind of defects and integral surfaces. If there exists an integral surface *A*_1_ that does not intersect with any defects’ domain, or in other words, the entire surface *A*_1_ is embedded within the ZIM, then Eq. (3a) will be satisfied. Conversely, any selected integral surface *A*_2_ must intersect with the defects’ domain; otherwise, Eq. (3b) cannot be fulfilled. These conditions on integral surfaces impose requirements on the defect connectivity inside the ZIM, that is, the defects must exhibit the long-range connectivity in the *z* direction, while generally remaining disconnected in the *x* direction to achieve CPA.

To fulfill the requirements on defect connectivity, the cylinder cluster depicted in Figure 2(a) establishes a continuous connection between the upper and lower PEC plates. Since the PEC plates can be viewed as mirror symmetry planes, they create mirror images of the connected cylinders. As a result, the long-range connectivity of cylinders is established along the *z* direction. This configuration ensures the necessary connectivity conditions for CPA realization.

To endow the ZIM slab with absorption capability, we assume the two thick dielectric cylinders to be dissipative, characterized by a complex relative permittivity *ɛ*_1_. In order to identify the parameter combinations leading to maximal absorption, we plot the absorptance as a function of the real part of *ɛ*_1_ (i.e., Re(*ɛ*_1_)) and imaginary part of *ɛ*_1_ (i.e., Im(*ɛ*_1_)), as presented in Figure 2(b). The relevant parameters are set as *l*_*x*_ = *l*_*y*_ = *l*_*z*_ = 2*λ*_0_, *r*_1_ = 0.3*λ*_0_, *l*_1_ = 0.9*λ*_0_, *r*_2_ = 0.03*λ*_0_, and *l*_2_ = 0.2*λ*_0_, where *λ*_0_ is the free-space wavelength, fixed in the numerical calculations. The methods for absorptance computation are presented in Supporting Information. We see that the absorption reaches near-100 % when *ɛ*_1_ = 9 + 5.5*i*, indicating successful realization of CPA. Importantly, the absorption remains significantly high as variations of *ɛ*_1_, signifying the robustness of the CPA. To validate the CPA behavior, we conduct numerical simulations using the finite-element software COMSOL Multiphysics. Figure 2(c) displays the simulated *E*_*z*_/*E*_0_ (color), time-averaged energy flux (arrows), and |**E**|/*E*_0_ (red line) along the white dashed line for the model with *ɛ*_1_ = 9 + 5.5*i* under the illumination of two counter-propagating planer waves. The *E*_*z*_/*E*_0_ profile shows the wave behavior in the whole model, in which strong electric field is observed inside the absorptive dielectric cylinders. On the other hand, from the |**E**|/*E*_0_ profile, constant electric field amplitude is observed in the air regions, indicating the disappearance of standing waves due to the interference of incident and reflected waves. Thus, we obtain near-zero reflection and almost perfect on the ZIM slab. In fact, both the incident waves are fully absorbed by the two thick dissipative cylinders, as evidenced by the energy flux distributions. These results conclusively demonstrate the occurrence of CPA in the 3D ZIM system.

It is noteworthy that the CPA is remarkably robust against the deformation of the embedded cylinders, as long as the long-range connectivity along the *z* direction is maintained. To demonstrate this robustness, we consider a lateral shift *d* in the *y* direction between the upper and lower thick cylinders, as well as a rotation angle *α* of the cylinder cluster measured from the *z* axis, as illustrated by the insets in Figure 2(d) and (e). The absorptance for both cases is presented in Figure 2(d) and (e), respectively, revealing robust near-perfect absorption. We further verify the robustness by simulating the *E*_*z*_/*E*_0_ (color), time-averaged energy flux (arrows), and |**E**|/*E*_0_ (red line) along the white dashed line when changing both the position and orientation of the cylinder cluster (Figure 2(f)). Notably, near-constant electric field amplitude is observed in the air regions, confirming the robustness of CPA based on 3D ZIM against the deformation of the embedded cylinders in the presence of long-range connectivity.

However, it is crucial to note that a minor disruption to this long-range connectivity will lead to a complete reduction of absorption, resulting in 100 % reflection and zero absorption of incident waves. Once the long-range connectivity along the *z* direction is broken for the model in Figure 2(a), we can always find out integral surfaces *A*_1_ and *A*_2_ that do not intersect with any cylinder. Hence, we have

(4a) $$\underset{\partial A_{1},D}{\oint }E\cdot \text{d}l=0,$$

and

(4b) $$\underset{\partial A_{2},D}{\oint }H\cdot \text{d}l=0.$$

**Figure 3:**
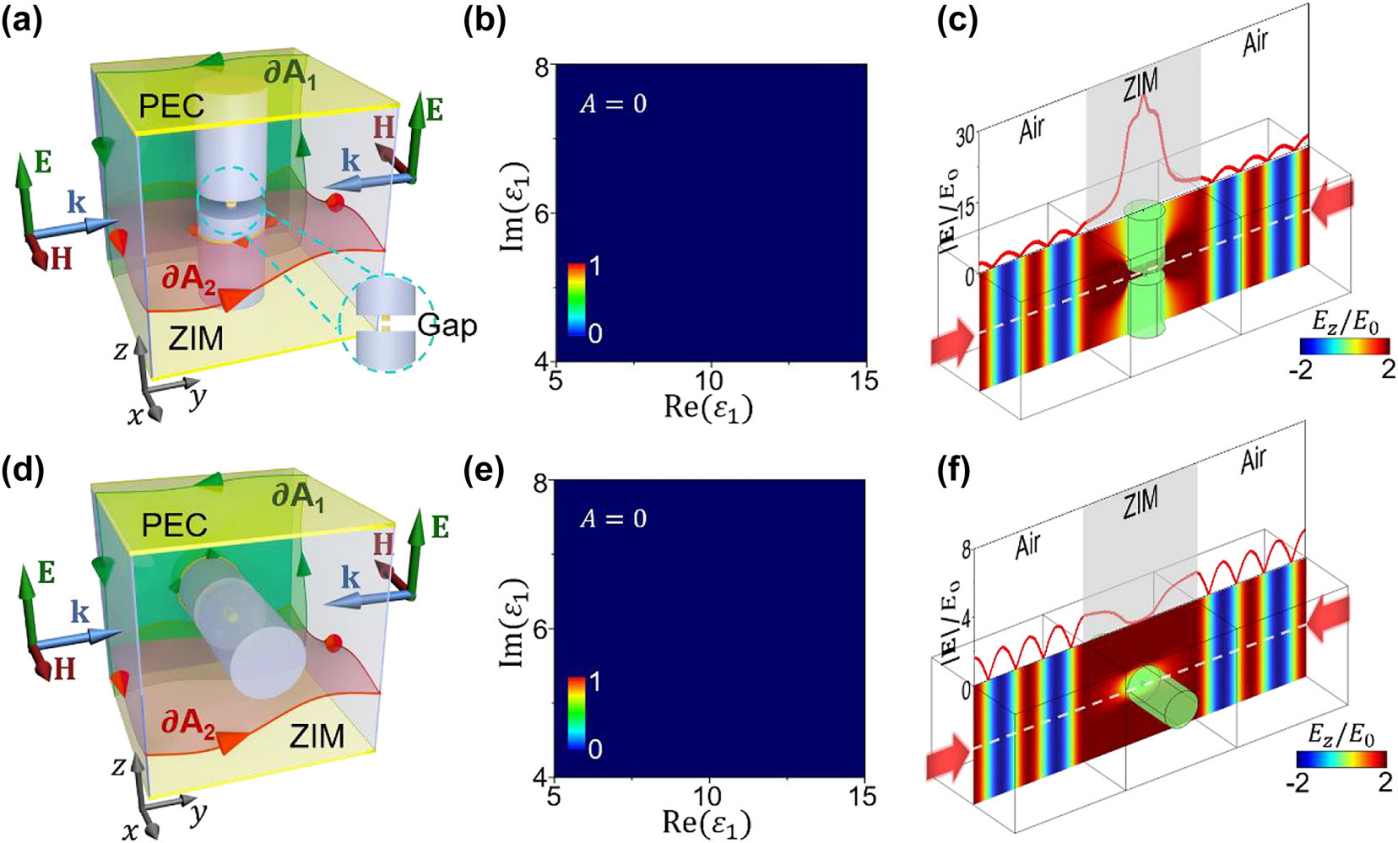
Zero absorption in the absence of long-range connectivity of defects in the vertical direction. (a) Schematic graph of the two-channel CPA model when the central thin PEC cylinder is cut to create a gap of 0.1*λ*_0_. (b) Absorptance with respect to Re(*ɛ*_*r*_) and Im(*ɛ*_*r*_) of the thick cylinders for the model in (a). (c) Simulated *E*_*z*_/*E*_0_ (color) and |**E**|/*E*_0_ (red line) along the white dashed line for the model in (a) with *ɛ*_*r*_ = 9 + 5.5*i*. (d) Schematic graph of the two-channel CPA model when the cylinder cluster is rotated to be oriented along the *x* direction. (e) Absorptance with respect to Re(*ɛ*_*r*_) and Im(*ɛ*_*r*_) of the thick cylinders for the model in (d). (f) Simulated *E*_*z*_/*E*_0_ (color) and |**E**|/*E*_0_ (red line) along the white dashed line for the model in (d) with *ɛ*_*r*_ = 9 + 5.5*i*.

Combining with Eq. (2), we obtain *R*_*L*_ = *R*_*R*_ = 1, indicating zero absorption in this case. As an example for verification, we cut the thin PEC cylinder to create a gap of 0.1*λ*_0_ (Figure 3(a)). The absorptance map displayed in Figure 3(b) shows completely zero absorption as the variations of *ɛ*_1_. This implies that the CPA is impossible in this scenario. In addition, we re-simulate the model in Figure 2(c), but with a gap of 0.1*λ*_0_ in the central thin PEC cylinder. From the simulation results, standing waves with normalized amplitudes oscillating from 0 to 2 in the air regions (|**E**|/*E*_0_ profile) and zero electric field inside the thick cylinders (*E*_*z*_/*E*_0_ profile) are observed, indicating 100 % reflection and zero absorption of incident waves, as further evidenced by the zero electric field within the thick cylinders.

In the second example, we rotate the cylinder cluster to be oriented along the *x* direction (Figure 3(d)). In this case, we have $\oint_{\partial A_{2},D}H\cdot \text{d}l=0$ . According to Eq. (2b), we obtain *R*_*R*_ + *R*_*L*_ = 2. This indicates that *R*_*L*_ = *R*_*R*_ = 1, also implying 100 % reflection of incident waves. As expected, zero absorption, irrespective of *ɛ*_1_, is observed in the absorptance map in Figure 3(e). This zero absorption property is further verified by the presence of standing waves in the air regions and zero electric fields inside the thick cylinders (Figure 3(f)). The above results demonstrate that the CPA based on 3D ZIM is crucially sensitive to the connectivity perturbations, which provides an ultra-sensitive approach for controlling CPA.

It is noteworthy that although above connectivity-sensitive CPA is demonstrated for transverse-electromagnetic (TEM) modes inside the PEC-PMC waveguide, the principle is also applicable for transverse-electric (TE) and transverse-magnetic (TM) modes. A TE or TM mode can be viewed as the superposition of two TEM modes propagating at the same oblique angle [16]. Through designing the ZIM slab with appropriately inclined surfaces, the connectivity-controlled CPA can also be realized for TE and TM waves (see Supporting Information).

### 2.2 Connectivity-controlled geometry-invariant multichannel CPA

**Figure 4:**
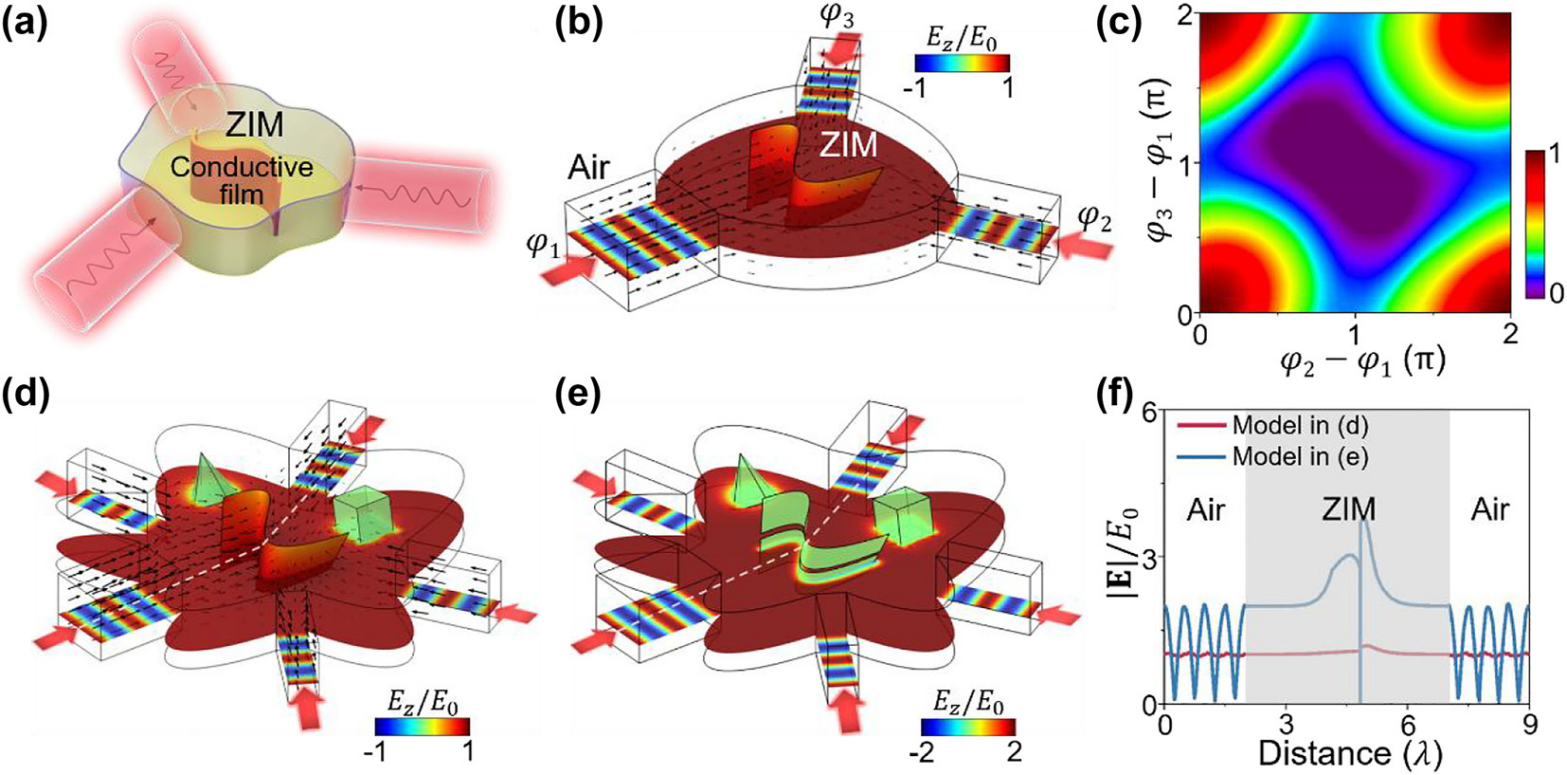
Connectivity-controlled geometry-invariant multichannel CPA. (a) Schematic graph of a three-channel CPA model consisting of an irregular 3D ZIM embedded with a curved conductive film. (b) Simulated *E*_*z*_/*E*_0_ (color) and time-averaged energy flux (arrows) for the three-channel model with *R*_*s*_ = 0.9*Z*_0_. (c) Absorptance as a function of the phase differences among the three channels in (b). [(d) and (e)] Simulated *E*_*z*_/*E*_0_ (color) and time-averaged energy flux (arrows) in a five-channel model when additional two defects are embedded inside the ZIM. The curved conductive film connects upper and lower PEC plates in (d), but is cut to create a gap of 0.1*λ*_0_ in (e). (f) Simulated |**E**|/*E*_0_ along the white dashed lines in the models in (d) and (e).

Superior to the traditional CPA of the two-channel configuration [4–6], the CPA based on 3D ZIM possesses a unique property of geometry-invariance and allows more than two input channels, offering additional degrees of freedom to control absorption. As a demonstration, we introduce a three-channel CPA model comprising an irregular 3D ZIM and a curved conductive film embedded within it (Figure 4(a)). The conductive film is designed to be ultrathin, with a thickness *t* much smaller than the waveguide *λ*_0_. This ultrathin film can be characterized by a sheet resistance *R*_*s*_, defined as 1/(*σ*_0_*t*) with *σ*_0_ being the conductivity, measuring the in-plane resistance for a film of arbitrarily sized square shape [44]. Alternatively, it can be approximately characterized by a relative permittivity of *iZ*_0_/(*k*_0_*tR*_*s*_) [44]. In practice, such a conductive film can be easily realized by a sheet of conductive materials like graphene and indium tin oxide at low frequencies [16, 19, 45]. The ZIM is positioned inside a PEC-PMC waveguide, consisting of upper and lower PEC plates and surrounding PMC plates. The embedded conductive film serves as a connecting link between the upper and lower PEC plates, establishing the long-range connectivity in the vertical direction. This configuration ensures the essential connectivity conditions for realizing CPA.

Figure 4(b) presents the simulation results for the three-channel model. In this configuration, three incident waves having the same phase and amplitude (with electric field polarized in the vertical direction) simultaneously impinge upon the ZIM from different directions. From the distributions of *E*_*z*_/*E*_0_ (color) and time-averaged energy flux (arrows), it is evident that all incident waves are absorbed by the embedded conductive film. Here, we set the sheet resistance of the conductive film as *R*_*s*_ = 0.9*Z*_0_. Actually, even when the *R*_*s*_ deviates from this value, near-perfect absorption can still be obtained. Then, to investigate the tuning capability of the three-channel model, we modify the phases of the three incident waves, denoted as *φ*_1_, *φ*_2_, and *φ*_3_, respectively. In Figure 4(c), we display the absorptance map as a function of the phase differences *φ*_2_ − *φ*_1_ and *φ*_3_ − *φ*_1_. It is seen that the perfect absorption occurs at a phase difference of 0 or 2*π*, and zero absorption occurs at a phase difference of *π*. By changing the phase difference either *φ*_2_ − *φ*_1_ or *φ*_3_ − *φ*_1_, the absorption can be tuned efficiently in an oscillatory manner.

Furthermore, introducing additional input channels allows for more degrees of freedom in controlling the absorption. We find that when dividing the three channels into more channels, while maintaining the total width of all channels unchanged, the required *R*_*s*_ remains unaffected. In Figure 4(d), we divide the three channels in Figure 4(b) into five channels while maintaining the total width unchanged. Additionally, two more defects are embedded within the ZIM, but these defects remain disconnected from the upper and lower PEC plates. These additional defects do not alter the connectivity characteristics of defects in this model. Furthermore, the size and shape of the ZIM are modified. Interestingly, we find that these changes do not affect the CPA behavior. The conductive film in Figure 4(d) is the same as that in Figure 4(b), and near-perfect absorption is achieved, as verified by the simulation results in Figure 4(d)–(f). These results demonstrate the realization of geometry-invariant multichannel CPA based on 3D ZIM, and highlight the robustness of CPA as long as the connectivity characteristics remain unchanged. However, it is important to note that if the long-range connectivity is disrupted, the absorption will immediately reduce to zero. As seen in Figure 4(e) and (f), when the conductive film is cut to create a gap of 0.1*λ*_0_, evident standing waves in the air regions are observed, indicating 100 % reflection and zero absorption of incident waves.

The above results demonstrate the intriguing properties of CPA based on 3D ZIM. Specifically, the CPA is highly robust against deformations of the embedded defects, changes in size and shape of the ZIM, as well as variations in number and orientation of incident channels, as long as the long-range connectivity is established. However, it is crucially sensitive to the perturbations to connectivity characteristics of defects. This connectivity-sensitive geometry-invariant multichannel CPA offers promising possibilities for advanced and flexible control of wave absorption.

### 2.3 Practical implementation based on 3D PhCs

In the following, we will demonstrate a practical implementation of the connectivity-controlled CPA based on 3D ZIM. The key to the successful design lies in achieving 3D ZIM with low loss. Fortunately, 3D PhCs exhibiting Dirac-like cone dispersion offers a platform for realizing the low-loss 3D ZIM [31, 34, 46].

**Figure 5:**
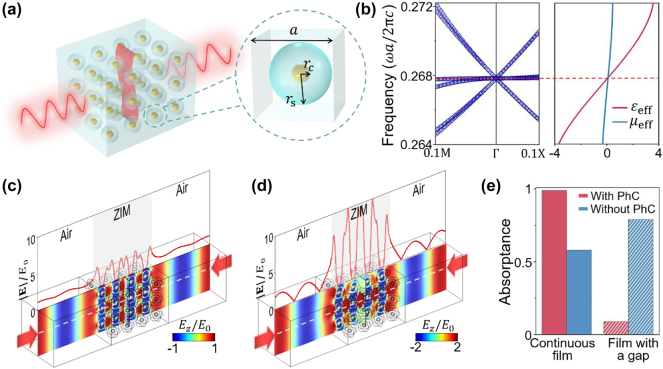
Practical implementation based on 3D PhCs. (a) A practical implementation a two-channel CPA using a 3D PhC-based ZIM. The PhC is composed of a simple cubic lattice of core–shell spheres. (b) Band structure of the PhC and its effective parameters *ɛ*_eff_ and *μ*_eff_ nearby the Dirac-point frequency. [(c) and (d)] Simulated *E*_*z*_/*E*_0_ (color) and |**E**|/*E*_0_ (red line) along the white dashed line under illumination of two counter-propagating waves. In (c), a strip of conductive film connecting the upper and lower boundaries is placed inside the PhC. In (d) the conductive film is cut to create a gap of *a*. (e) Absorptance for the models in (c) and (d) in the presence or absence of the PhC.

Here, we make use of a PhC composed of a simple cubic lattice of core–shell spheres with a lattice constant of *a* (Figure 5(a)). The core of the sphere is made of PEC (radius *r*_c_ = 0.126*a*), and the shell is made of a dielectric material (radius *r*_s_ = 0.4*a*, relative permittivity *ɛ*_s_ = 30). We note that this high-index dielectric material can be realized in the microwave regime [47]. In Figure 5(b), the left panel graph shows a Dirac-like cone at the center of the Brillouin zone (i.e., the Γ point) as the consequence of sixfold degenerate modes occurring at the normalized frequency of *ωa*/2*πc* = 0.2678. Previous researches have demonstrated that at the Dirac-point frequency, the PhC effectively works as a uniform ZIM possessing vanishing permittivity and permeability simultaneously [31, 34, 46, 48], [49], [50]. The right panel graph shows the effective relative permittivity *ɛ*_eff_ and relative permeability *μ*_eff_ of this PhC, both of which are found to be zero at the Dirac-point frequency. Here, the effective parameters are retrieved based on the averaged eigen-fields [34, 51].

By utilizing this 3D PhC-based ZIM, we construct a two-channel CPA model comprising 3 × 3 × 4 PhC units, strategically arranged as depicted in Figure 5(a). Within the PhC, an ultrathin conductive film strip (thickness 0.02*a*, width *a*, sheet resistance 0.2*Z*_0_) connects the upper and lower surfaces, thus establishing the long-range connectivity in the vertical direction. Figure 5(c) presents the simulated *E*_*z*_/*E*_0_ (color) and |**E**|/*E*_0_ (red line) along the white dashed line under the illumination of two counter-propagating waves at the Dirac-point frequency. The nearly constant electric field amplitude observed in the air regions indicates the near-perfect absorption of incident waves. We find that the absorptance reaches an impressive value of 0.99 (Figure 5(e)). Next, we introduce a gap of *a* by cutting the conductive film. From the simulation results in Figure 5(d), a substantial amount of reflection is observed, leading to a significant reduction in absorptance to 0.09 (Figure 5(e)). This clearly shows the connectivity-controlled absorption behavior. For comparison, we remove the PhC, as shown in Figure 5(d) and (e). It is seen that the CPA disappears, and the sensitivity on the connectivity perturbations is diminished. In Supporting Information, the connectivity-controlled CPA is further demonstrated based on an alternative design, i.e., all-dielectric PhCs, which could be realized at higher frequencies. Overall, the proposed implementation using 3D PhC-based ZIM has shown the feasibility and effectiveness in realizing and controlling CPA through engineering the defect connectivity.

## 3 Discussion and conclusion

Compared to the traditional CPA of the two-channel configuration [4–6], the implementation of CPA based on 3D ZIM offers the advantage of accommodating more than two input channels. This feature provides additional degrees of freedom to efficiently control absorption. Notably, our proposed approach introduces a critical role for defect connectivity within the ZIM-based CPA, beyond the phase differences among different incident waves, further enhancing control capabilities. Our approach opens up promising avenues for advanced CPA control with versatile functionalities.

It is important to highlight that the underlying physics governing the CPA using 2D and 3D ZIM is fundamentally different. In 2D ZIM-based CPA [22, 23], the principle relies on the photonic doping effect [37–42], which however, is absent in 3D ZIM due to distinct electromagnetic wave behaviors [13, 33, 34]. The electromagnetic waves in 2D ZIM follow scalar wave equations, while they obey vector wave equations in 3D ZIM. Consequently, in 2D ZIM-based CPA, variations in geometrical and electromagnetic parameters (e.g., size, shape, and permittivity) significantly affect the absorption properties due to the photonic doping effect [22, 23]. Nevertheless, in 3D ZIM-based CPA, the absorption is primarily governed by the establishment of long-range connectivity among defects, rather than their details. As long as the long-range connectivity of defects is established, the CPA exhibits remarkable robustness against variations in the defects’ geometrical and electromagnetic parameters.

In conclusion, we have highlighted the essential role of defect connectivity within 3D ZIM for realizing and controlling the CPA. The intriguing properties of 3D ZIM-based CPA have been elucidated, revealing its sensitivity to perturbations in defect connectivity, while demonstrating remarkable robustness against deformations of defects, changes in size and shape of the ZIM, and variations in number and orientation of incident channels, as long as the long-range connectivity is maintained. Furthermore, geometry-invariant multichannel CPA based on 3D ZIM, as well as the practical implementation using PhCs exhibiting a Dirac-like cone dispersion have been demonstrated. These findings open up exciting possibilities for advanced and flexible control of absorption with potential applications in various fields, such as sensors, modulators, and switches.

## Supplementary Material

Supplementary Material Details
