# Supplementary material for: Controlling coherent perfect absorption via long-range connectivity of defects in three-dimensional zero-index media
Source: Nanophotonics. 2023 Oct 25;12(22):4195–204. doi: 10.1515/nanoph-2023-0485 (PMC11501844; doi:10.1515/nanoph-2023-0485)
Supplement: Supplementary file 1 — Supplementary Material Details [file j_nanoph-2023-0485_suppl_001.docx]

Supporting Information

**Controlling coherent perfect absorption via long-range connectivity of defects in three-dimensional zero-index media**

*Dongyang Yan, Ran Mei, Mingyan Li, Zhikai Ma, Zhi Hong Hang^*^, and Jie Luo^*^*

Dongyang Yan, Ran Mei, Mingyan Li

Institute of Theoretical and Applied Physics, School of Physical Science and Technology, Soochow University, Suzhou 215006, China

Zhikai Ma

State Key Laboratory for Mesoscopic Physics and Frontiers Science Center for Nano-optoelectronics, School of Physics, Peking University, Beijing 100871, China.

Zhi Hong Hang

School of Physical Science and Technology and Collaborative Innovation Center of Suzhou Nano Science and Technology, Soochow University, Suzhou 215006, China

Institute for Advanced Study, Soochow University, Suzhou 215006, China

Provincial Key Lab of Thin Films, Soochow University, Suzhou 215006, China

E-mail: [zhhang@suda.edu.cn](mailto:zhhang@suda.edu.cn)

Jie Luo

Institute of Theoretical and Applied Physics, School of Physical Science and Technology, Soochow University, Suzhou 215006, China

Provincial Key Lab of Thin Films, Soochow University, Suzhou 215006, China

E-mail: [luojie@suda.edu.cn](mailto:luojie@suda.edu.cn)

1. **Methods for absorptance computation**
2. **Connectivity-controlled CPA for TE modes**
3. **Practical implementation based on all-dielectric PhCs**

# Methods for absorptance computation

In numerical simulations, we employed two methods to compute the absorptance. The first method is to calculate the absorptance according to its definition, that is, the ratio of the energy absorbed by a body to that incident upon it. For a $N$-channel model, the absorptance $A$ can be expressed as,

$A=1-\left| \frac{\sum_{n=1}^{N} \iint\left\langle\mathbf{P}_{\mathrm{out},n} \right\rangle\cdot d\mathbf{S}_{n}}{\sum_{n=1}^{N} \iint\left\langle\mathbf{P}_{\mathrm{in},n} \right\rangle\cdot d\mathbf{S}_{n}} \right|$, (S1)

where $\left\langle\mathbf{P}_{\mathrm{out},n} \right\rangle$ and $\left\langle\mathbf{P}_{\mathrm{in},n} \right\rangle$ denote time-averaged energy flux density for the outgoing and incident waves in the $n$-th channel, respectively. Figure S1(a) shows a specific example based on the finite-element software COMSOL Multiphysics, by using which the distributions of time-averaged energy flux density can be directly obtained. Then, we choose a cross section of each channel as the integral surface (marked in Fig. S1(a)) to compute the integrals $\iint\left\langle\mathbf{P}_{\mathrm{out},n} \right\rangle\cdot d\mathbf{S}_{n}$ and $\iint\left\langle\mathbf{P}_{\mathrm{in},n} \right\rangle\cdot d\mathbf{S}_{n}$. Finally, we can obtain the absorptance of this model according to Eq. (S1).


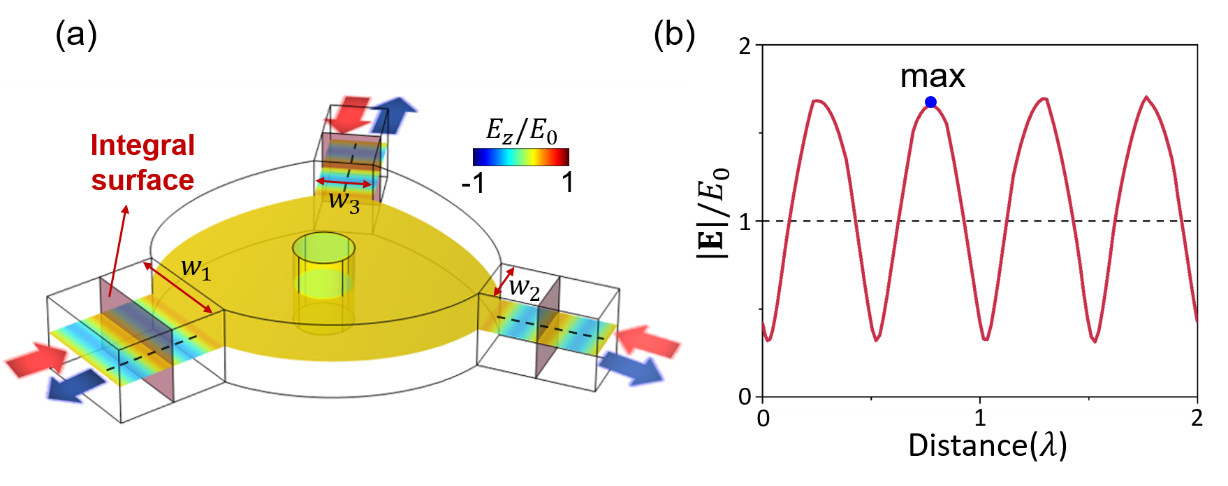


**Figure S1.** (a) A three-channel model to illustrate the absorptance computation in numerical simulations. The cross sections of incident channels are the integral surfaces. (b) Normalized electric field amplitude in the channel along the black dashed lines.

Alternatively, we can evaluate the absorptance through analyzing the amplitude of standing waves in incident channels. Considering that electromagnetic waves in all channels are transverse electromagnetic (TEM) modes, we have $\left\langle\mathbf{P}_{\mathrm{in},n} \right\rangle=\frac{E_{0}^{2}}{2Z_{0}}\mathbf{e}_{k}$ and $\left\langle\mathbf{P}_{\mathrm{out},n} \right\rangle=\frac{\left| r_{n} \right|^{2}E_{0}^{2}}{2Z_{0}}\mathbf{e}_{k}$. Here, $E_{0}$ is the electric field amplitude of incidence, which is the same for all incident channels. $r_{n}$ is the reflection coefficient in the $n$-th channel, which is defined as the ratio of the electric field of the outgoing waves to that of the incident waves. $Z_{0}$ is the impedance of free space. Since the electric field amplitude of the incidence is the same for all incident channels, Eq. (S1) can be simplified to

$A=1-\sum_{n=1}^{N} \frac{\left| r_{n} \right|^{2}w_{n}}{W}$, (S2)

where $w_{n}$ is the width of the $n$-th channel. $W$ ($=\sum_{n=1}^{N} w_{n}$) is the total width of all channels. Then, we analyze the standing waves in the incident channels to evaluate the reflection coefficient $r_{n}$. As an example, in Fig. S1(b) we plot the normalized electric field amplitude in the incident channel along the black dashed lines in the model in Fig. S1(a). Considering that the standing waves are the result of superposition of the incident and reflected waves, the maximal value of the electric field amplitude is $(1+r_{n})E_{0}$. Therefore, through identifying the maximum value of the electric field amplitude $E_{\max,n}$ in Fig. S1(b), the reflection coefficient of the $n$-th channel $r_{n}$ can be expressed as,

$r_{n}=\frac{E_{\max,n}}{E_{0}}-1$. (S3)

By inserting Eq. (S3) into Eq. (S2), we obtain

$A=1-\sum_{n=1}^{N} \frac{\left| \frac{E_{\max,n}}{E_{0}}-1 \right|^{2}w_{n}}{W}$. (S4)

Equation (S4) establishes the relationship between the absorptance and the amplitude of standing waves in incident channels. In our numerical calculations, we have employed both Eqs. (S1) and (S4) to compute the absorptance, which is found to be the same.

# Connectivity-controlled CPA for TE modes

Usually, the zero-index media (ZIM) are only applicable for normally incident TEM waves [1, 2]. Interestingly, here we find that our model also works for transverse-electric (TE) and transverse-magnetic (TM) modes when the ZIM possess inclined surfaces. This is because the TE and TM modes can be viewed as the superposition of two TEM modes propagating at the same oblique angle [3], as illustrated in Fig. S2(a). Thus, it is possible to realize the connectivity-controlled CPA for TE and TM modes when using ZIM with appropriate inclined surfaces. In the following, we’d like to show an example for TE modes.


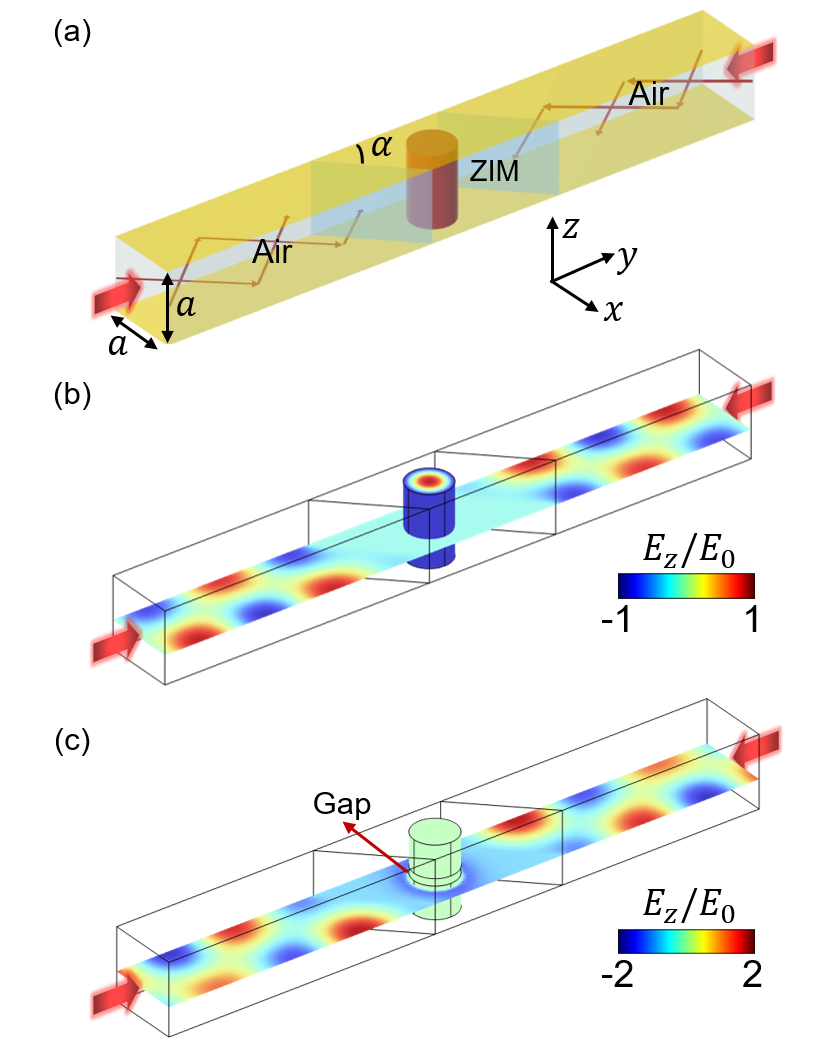


**Figure S2.** (a) Schematic graph of a two-channel CPA model in a PEC-PMC waveguide for TE modes. The PEC-PMC waveguide consists of upper and lower PEC plates and side PMC plates. Two counter-propagating TE waves are incident onto the central ZIM slab with inclined surfaces. Inside the ZIM, a cylindrical defect is vertically placed, connecting the upper and lower PEC plates. The TE mode can be viewed as the superposition of two TEM modes propagating at the same oblique angle. (b) Simulated $E_{z}/E_{0}$ in the case of CPA under the illumination of two counter-propagating TE_10_ modes. (c) Simulated $E_{z}/E_{0}$ when the cylindrical defect is cut to create a gap of $0.1a$.

Figure S2(a) shows the schematic graph of a two-channel CPA model in a PEC-PMC waveguide. Two counter-propagating TE_10_ mode are incident onto the central ZIM slab with inclined surfaces. Inside the ZIM, a cylindrical defect is vertically placed, connecting the upper and lower PEC plates. The TE_10_ mode is characterized by the electric field of

$\mathbf{E}=E_{0}\cos(k_{x}a)e^{-i\omega t\pm ik_{y}y}\hat{\boldsymbol{z}}$, (S5)

where $k_{x}=\pi/a$ and $k_{y}=\sqrt{k_{0}^{2}-k_{x}^{2}}$. $k_{0}$ is the wave number in free space. $a$ is the separation distance of the two PMC plates, which are located on the planes of $x=0$ and $x=a$, respectively. Such a TE_10_ mode can be viewed as the superposition of two TEM modes propagating at the same oblique angle of

$\theta_{0}=\cos^{-1} (k_{y}/k_{0})$. (S6)

Here, we choose the working wavelength as $\sqrt{2}a$, then we get $\theta_{0}=45^{\circ}$ according to Eq. (S6). Accordingly, the inclined angle of the ZIM’s inclined surfaces are set as $\alpha=45^{\circ}$. Figure S2(b) shows the simulated $E_{z}/E_{0}$ under the illumination of two counter-propagating TE_10_ modes. Here, the radius and relative permittivity of the cylindrical defect are set as $0.3a$ and $9+2.5i$, respectively. We see that the normalized electric field amplitude in the air regions is near unity, indicating the occurrence of CPA. Through numerical calculation, we find that the absorptance is as high as 0.99. Interestingly, the absorptance drops rapidly to near zero when the cylindrical defect is cut to create a gap of $0.1a$, as shown in Fig. S2(c). These results demonstrate that the connectivity controlled CPA can also be realized for TE modes. This principle also applies to TM modes.

# Practical implementation based on all-dielectric PhCs

In this section, we show a scheme for three-dimensional (3D) ZIM by using an all-dielectric photonic crystal (PhC). The model is adopted from [2]. The unit cell of the 3D dielectric PhC is illustrated in Fig. S3(a), which has a cubic lattice with lattice constant $a$. Three identical dielectric meshes (relative permittivity 40) orthogonally intersect at the center of the unit cell. The cross sections of the meshes are squares with the side length $l=0.343a$. Figure S3(b) presents the band structure of this PC in the first Brillouin zone (left), showing a Dirac-like cone as the consequence of sixfold accidental degeneracy of electric and magnetic dipolar states at the Γ point. At the Dirac-point frequency, the PhC effectively serves as a 3D ZIM, as evidenced by the effective parameters [right panel graph of Fig. S3(b)].


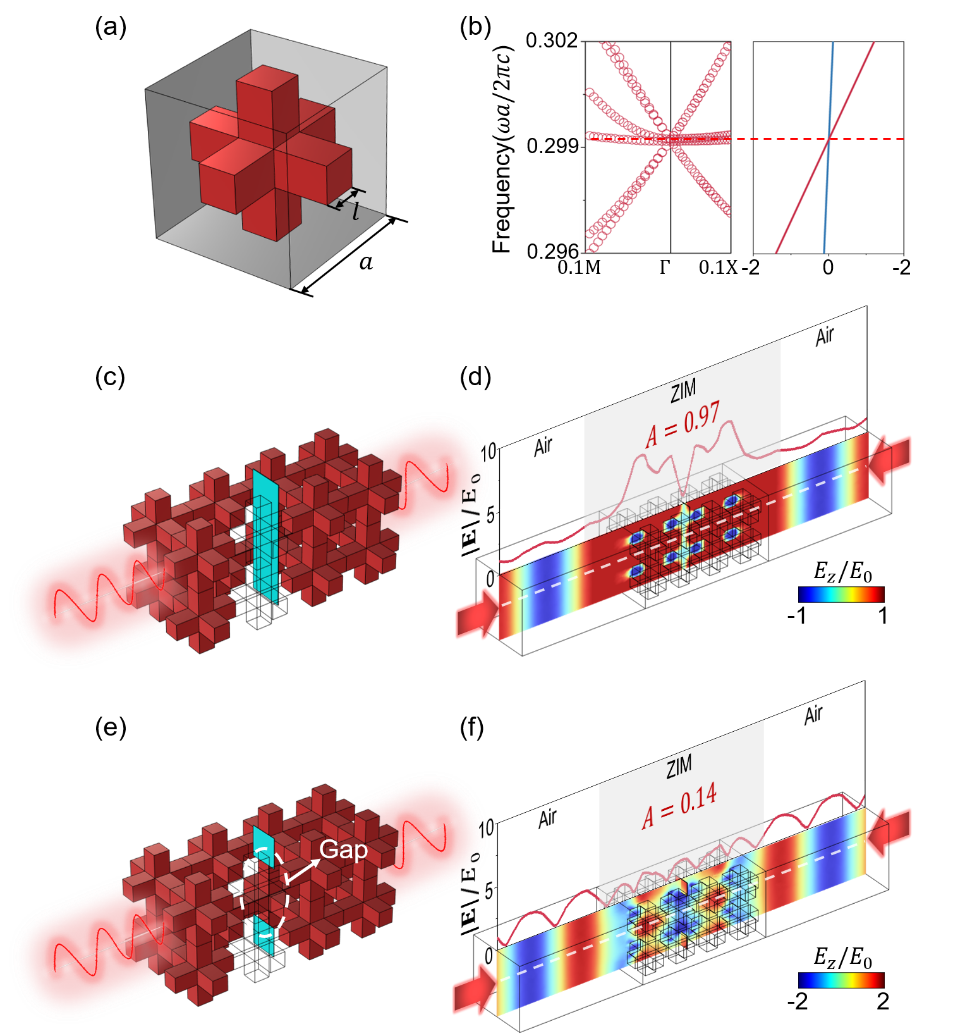


**Figure S3.** (a) The unit cell of an all-dielectric PhC consisting of three orthogonally intersected identical dielectric meshes. (b) Band structure of the PhC and its effective parameters $\varepsilon_{\mathrm{eff}}$ and $\mu_{\mathrm{eff}}$ nearby the Dirac-point frequency. [(c) and (e)] Schematic graphs of CPA configurations using the dielectric PhC. In (c) a strip of conductive film connecting the upper and lower boundaries is placed inside the PhC. In (e) the conductive film is cut to create a gap of $1.2a$. [(d) and (f)] Simulated $E_{z}/E_{0}$ (color) and $|\mathbf{E}|/E_{0}$ (red line) along the white dashed line under illumination of two counter-propagating waves, corresponding to the models in (c) and (e), respectively.

By utilizing this 3D PhC-based ZIM, we construct a two-channel CPA model comprising $2\times2\times4$ PhC units, strategically arranged as depicted in Fig. S3(c). Within the PhC, an ultrathin conductive film strip (thickness $0.02a$, width $0.5a$, sheet resistance $0.1Z_{0}$) connects the upper and lower surfaces, thus establishing the long-range connectivity in the vertical direction. Fig. S3(d) presents the simulated $E_{z}/E_{0}$ (color) and $|\mathbf{E}|/E_{0}$ (red line) along the white dashed line under the illumination of two counter-propagating waves at the Dirac-point frequency. We find that the absorptance is high (~0.97), as evidenced by the nearly constant electric field amplitude in the air regions. Interestingly, when the conductive film is cut to create a gap of $1.2a$ [Fig. S3(e)], a significant reduction in absorptance to 0.14 is observed [Fig. S3(f)]. These results clearly show the connectivity-controlled absorption behavior, and demonstrate the feasibility and effectiveness in realizing and controlling CPA using the dielectric PhC-based ZIM.

**References**

[1] X. Huang, Y. Lai, Z. H. Hang, H. Zheng, and C. T. Chan, “Dirac cones induced by accidental degeneracy in photonic crystals and zero-refractive-index materials,” Nat. Mater., vol. 10, pp. 582-586, 2011.

[2] C. Xu, H. Chu, J. Luo, Z. H. Hang, Y. Wu, and Y. Lai, “Three-dimensional electromagnetic void space,” Phys. Rev. Lett., vol. 127, pp. 123902, 2021.

[3] S. Li, J. Luo, S. Anwar, S. Li, W. Lu, Z. H. Hang, Y. Lai, B. Hou, M. Shen, and C. Wang, “Broadband perfect absorption of ultrathin conductive films with coherent illumination: Superabsorption of microwave radiation,” Phys. Rev. B, vol. 91, pp. 220301(R), 2015.
